# Caspase-Dependent Cleavage of DDX21 Suppresses Host Innate Immunity

**DOI:** 10.1128/mBio.01005-21

**Published:** 2021-06-14

**Authors:** Wei Wu, Yang Qu, Shengqing Yu, Sa Wang, Yuncong Yin, Qinfang Liu, Chunchun Meng, Ying Liao, Zaib Ur Rehman, Lei Tan, Cuiping Song, Xusheng Qiu, Weiwei Liu, Chan Ding, Yingjie Sun

**Affiliations:** a Department of Avian Infectious Diseases, Shanghai Veterinary Research Institute, Chinese Academy of Agricultural Science, Shanghai, People’s Republic of China; b College of Veterinary Medicine, Northwest A&F University, Yangling, Shaanxi, People’s Republic of China; c College of Animal Sciences, Fujian Agriculture and Forestry University, Fuzhou, People’s Republic of China; d Jiangsu Co-innovation Center for Prevention and Control of Important Animal Infectious Diseases and Zoonoses, Yangzhou, People’s Republic of China; University of KwaZulu-Natal

**Keywords:** DDX21, virus infection, cleavage, innate immunity

## Abstract

DEAD (Glu-Asp-Ala-Glu) box RNA helicases have been proven to contribute to antiviral innate immunity. The DDX21 RNA helicase was identified as a nuclear protein involved in rRNA processing and RNA unwinding. DDX21 was also proven to be the scaffold protein in the complex of DDX1-DDX21-DHX36, which senses double-strand RNA and initiates downstream innate immunity. Here, we identified that DDX21 undergoes caspase-dependent cleavage after virus infection and treatment with RNA/DNA ligands, especially for RNA virus and ligands. Caspase-3/6 cleaves DDX21 at D126 and promotes its translocation from the nucleus to the cytoplasm in response to virus infection. The cytoplasmic cleaved DDX21 negatively regulates the interferon beta (IFN-β) signaling pathway by suppressing the formation of the DDX1-DDX21-DHX36 complex. Thus, our data identify DDX21 as a regulator of immune balance and most importantly uncover a potential role of DDX21 cleavage in the innate immune response to virus.

## INTRODUCTION

The host innate immune response is initiated by virus infection. Several pathogen recognition receptors (PRRs) are mobilized to sense viral nucleic acids and ultimately lead to the induction of interferons (IFNs) and other inflammatory cytokines to protect host cells (1). Among them, endosomal Toll-like receptor 3 (TLR3) (2, 3), cytoplasmic retinoic acid-inducible gene I (RIG-I), and melanoma differentiation-associated protein 5 (MDA5) (4, 5) were demonstrated to be critical for sensing viral double-stranded RNA (dsRNA). The adaptor protein TIR domain-containing adaptor inducing interferon beta (TRIF) (2) and the mitochondrial protein MAVS (VISA, IPS-1, or Cardif) (6–9) are then activated, leading to the activation of nuclear factor kappa B (NF-κB) and the transfection factor interferon regulatory factor 3 (IRF3) as well as the production of various cytokines, including type I IFNs (10). The secreted IFN binds to the IFN receptor and induces the expression of various interferon-stimulated genes (ISGs) to establish a cellular antiviral state (1).

The helicase family is a class of enzymes that are essential to all living organisms. Their main function is separating nucleic acid strands (DNA, RNA, or RNA-DNA hybrids) (11). The human genome encodes 64 RNA helicases and 31 DNA helicases, which are classified into several superfamilies (SFs) based on their conserved motifs (12, 13). The DEAD (Glu-Asp-Ala-Glu)/H box helicase belongs to SF2, the largest group of helicases, which are involved in various cellular processes (12, 13). RNA helicases are critical for most RNA metabolism processes and are also involved in the antiviral immune response by sensing foreign RNAs (14). RIG-I, MDA5, and Laboratory of Genetics and Physiology 2 (LGP2), three RIG-I-like receptors (RLRs) that belong to the SF2 RNA helicases, are closely related to DEAD box helicases (15). In addition to RLRs, a growing list of DEAD/H box helicases has been identified to contribute to antiviral innate immunity in recent years, by acting as either sensors for viral nucleic acids or mediators of downstream signaling events (16–21).

DDX21 was shown to be an abundant nuclear protein in HeLa cells that directly binds rRNAs and snoRNAs and promotes rRNA transcription, processing, and modification (22–25). Another important function of DDX21 is unwinding RNAs, including dsRNA and RNA guanine quadruplexes (26–28). Recently, several reports indicated that DDX21 also plays a role in innate immunity and virus infection. DDX21, together with DDX1 and DHX36, can bind the adaptor protein TRIF to sense dsRNA (20). During dengue virus infection, DDX21 translocates from the nucleus to the cytoplasm and mediates innate immunity (29). Moreover, DDX21 inhibits influenza A virus replication but is counteracted by viral NS1 protein (30). However, how DDX21 precisely regulates antiviral innate immunity and whether DDX21 undergoes protein modification during virus infection remain unclear.

Our preliminary screening results showed that Newcastle disease virus (NDV) manipulates the expression of several DEAD/H-box proteins (DDXs/DHXs) during infection (data not shown). Among them, DDX21 was found to be cleaved in HeLa cells infected with NDV. Here, we report that virus infection and RNA/DNA ligands cleave DDX21 at D126 via the caspase-3/6 pathway. The cleavage of DDX21 promotes its translocation from the nucleus to the cytoplasm in response to virus infection. The cytoplasmic cleaved DDX21 (cDDX21) negatively regulates the IFN-β signaling pathway by suppressing the formation of the DDX1-DDX21-DHX36 complex. Our study therefore reveals a role of DDX21 in the regulation of antiviral innate immunity and provides molecular insights into how the host balances antiviral and aberrant innate immunity.

## RESULTS

### DDX21 positively regulates the IFN-β signaling pathway.

**FIG 1:**
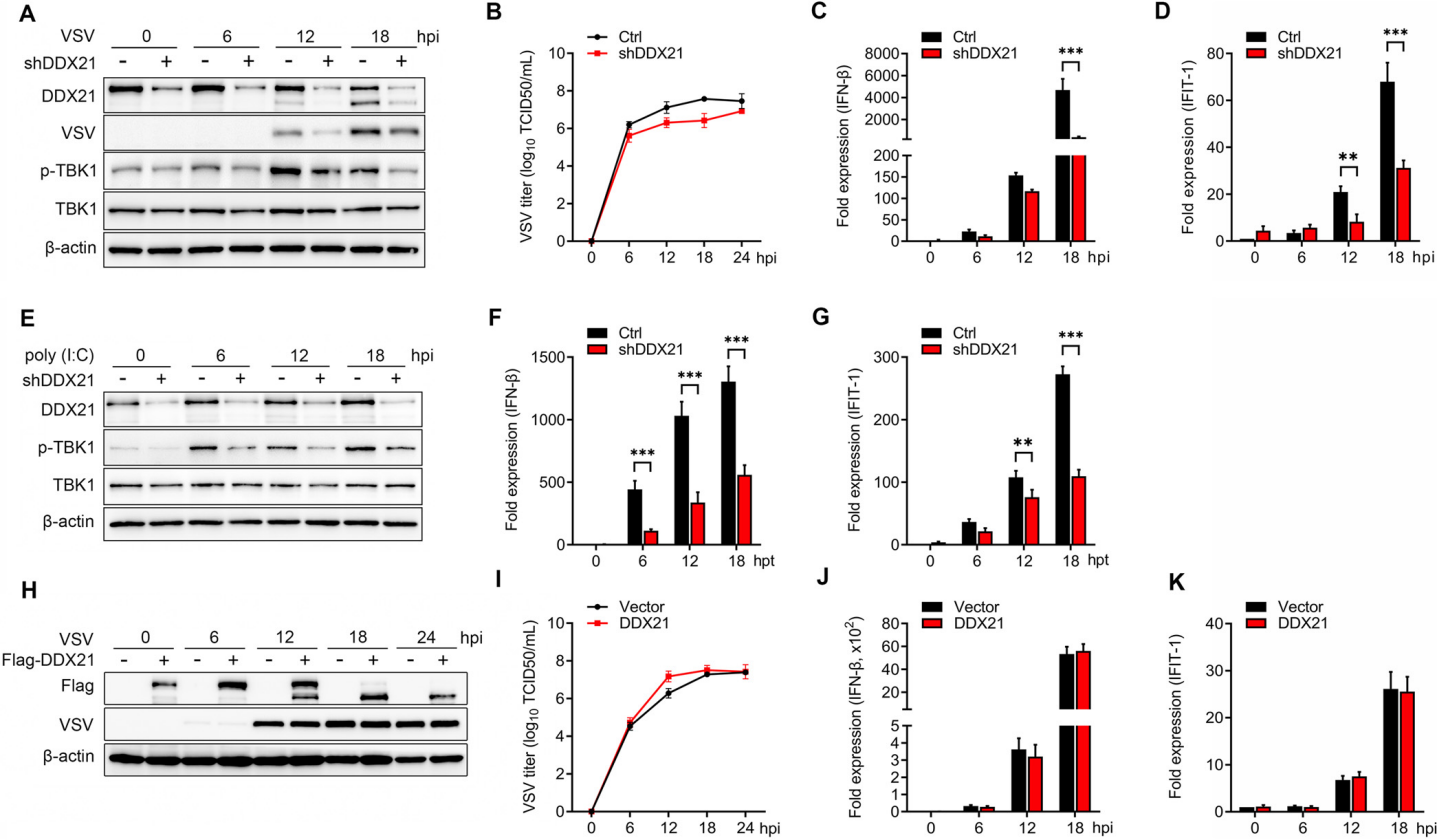
DDX21 positively regulates the IFN pathway. (A) HeLa cells with stable knockdown of DDX21 and control cells were mock treated or infected with VSV at a multiplicity of infection (MOI) of 1. Cells were harvested at 6, 12, 18, and 24 hpi and detected by immunoblot analysis with anti-DDX21, anti-VSV-G, anti-p-TBK1, anti-TBK1, or anti-β-actin antibody. (B) Extracellular virus yields in DDX21 knockdown and control cells. (C and D) Virus infection experiments were performed as described above for panel A. Cells were harvested and detected using qRT-PCR with IFN-β (C) and IFIT-1 (D) primers. (E) HeLa cells with stable knockdown of DDX21 and control cells were mock treated or transfected with poly(I·C) (20 μg/ml). Cells were harvested at 6, 12, and 18 hpt and detected by immunoblot analysis with anti-DDX21, anti-VSV-G, anti-p-TBK1, anti-TBK1, or anti-β-actin antibody. (F and G) Poly(I·C) treatment experiments were performed as described above for panel E. Cells were harvested and detected by qRT-PCR with IFN-β (F) and IFIT-1 (G) primers. (H) HeLa cells were transfected with either an empty vector or Flag-DDX21. Twenty-four hours after transfection, cells were mock treated or infected with VSV at an MOI of 1. Cells were harvested at 6, 12, 18, and 24 hpi and detected by immunoblot analysis with anti-DDX21, anti-VSV-G, or anti-β-actin antibody. (I) Extracellular virus yields in the empty vector- or Flag-DDX21-transfected group. (J and K) Virus infection experiments were performed as described above for panel G. Cells were harvested and detected by qRT-PCR with IFN-β (J) and IFIT-1 (K) primers. Data are presented as means from three independent experiments. *, *P* < 0.05; **, *P* < 0.01; ***, *P* < 0.001.

To confirm the role of DDX21 in virus replication as well as its role in antiviral innate immunity, DDX21 was knocked down, followed by vesicular stomatitis virus (VSV) infection. The results showed that the knockdown of DDX21 significantly reduced the expression of the VSV G protein (VSV-G) at 12, 18, and 24 h postinfection (hpi) (Fig. 1A). Consistently, the virus titers were significantly impaired after DDX21 knockdown at 6, 12, and 18 hpi (Fig. 1B). To study the role of DDX21 in innate immunity, the expression levels of IFN-β and the downstream interferon-stimulated gene (ISG) interferon-induced protein with tetratricopeptide repeats 1 (IFIT-1) were evaluated. The results showed that the knockdown of DDX21 inhibited the mRNA levels of IFN-β (Fig. 1C) and IFIT-1 (Fig. 1D). Consistently, the phosphorylation of TANK binding kinase 1 (TBK1) was significantly inhibited at 6, 12, and 18 hpi. Similar results were observed in DDX21 knockdown cells after treatment with poly(I·C), confirming the positive regulation of the IFN-β pathway by DDX21 (Fig. 1E to G). For further confirmation, we generated DDX21 knockout cells. Unfortunately, after three rounds of screening, only 1/225 heterozygous clones (*ddx21^+/−^*) was identified by sequencing (see Fig. S1A in the supplemental material). Western blotting showed that compared with wild-type (WT) cells, DDX21 expression was significantly inhibited in *ddx21*^+/−^ cells (Fig. S1B). The regulation of virus titers and the IFN-β pathway was in accord with the knockdown results (Fig. S1C to G). However, using the overexpression model, we observed that transfection of exogenous DDX21 increased VSV titers only at 12 hpi (Fig. 1I), and the synthesis of virus G protein was unchanged at all time points postinfection (Fig. 1H). Correspondingly, DDX21 overexpression had no effect on IFN-β production or the mRNA levels of ISGs (Fig. 1J and K). Collectively, these results suggested that DDX21 positively regulates the IFN-β signaling pathway. However, the discrepancy between the knockdown and overexpression models raised the question of whether, in addition to DDX21 expression, the modification of DDX21 plays a role in antiviral innate immunity.

10.1128/mBio.01005-21.1 FIG S1 DDX21 knockout inhibits the IFN signaling pathway. (A) Confirmation of genome editing by sequencing of the PCR amplicon from the DDX21 genome of the cell lines. (B) WT and *ddx21^+/−^* HeLa cells were seeded in 6-well plates and collected for immunoblot analysis with anti-DDX21 or anti-β-actin antibody. (C) WT and *ddx21^+/−^* HeLa cells were mock treated or infected with VSV at an MOI of 1. Cells were harvested at 6, 12, and 18 hpi and detected by immunoblot analysis with anti-DDX21, anti-VSV-G, or anti-β-actin antibody. (D) Extracellular virus yields in DDX21 knockdown and control cells. (E to G) Virus infection experiments were performed as described above for panel C. Cells were harvested and detected by qRT-PCR with IFN-β (E), IFIT-1 (F), and MX1 (G) primers. Download FIG S1, TIF file, 1.9 MB. Copyright © 2021 Wu et al. 2021 Wu et al. https://creativecommons.org/licenses/by/4.0/ This content is distributed under the terms of the Creative Commons Attribution 4.0 International license.

### Cleavage of DDX21 by virus infection and treatment with RNA/DNA ligands.

**FIG 2:**
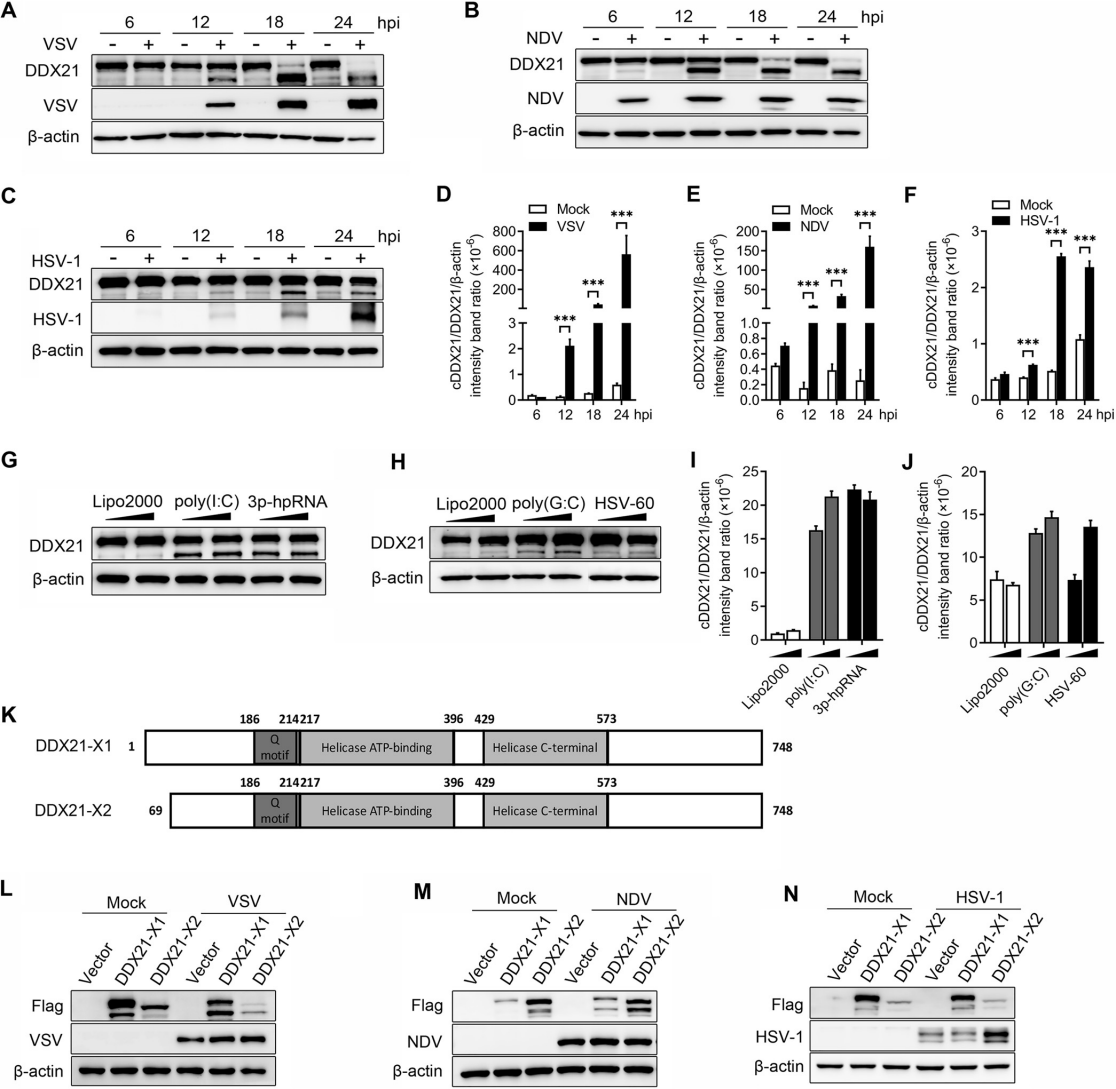
Virus infection or treatment with RNA/DNA ligands leads to the cleavage of DDX21. (A to C) HeLa cells were mock treated or infected with VSV (A), NDV (B), or HSV-1 (C) at an MOI of 1. Cells were harvested at 6, 12, 18, and 24 hpi and detected by immunoblot analysis with anti-DDX21, anti-β-actin, or anti-viral-protein (VSV-G, NDV-NP, or HSV-1-gD) antibody. (D to F) Representative results, with graphs representing the band intensity ratios of cleaved DDX21 (cDDX21)/DDX21/β-actin normalized to the control conditions for the VSV (D), NDV (E), and HSV-1 (F) infection groups. Data are presented as means from three independent experiments. ***, *P* < 0.001. (G and H) HeLa cells were transfected with RNA ligands [poly(I·C) or 3p-hpRNA] (G) or DNA ligands [poly(G·C) or HSV-60] (H). At 18 h posttransfection (hpt), cells were harvested and detected by immunoblot analysis with anti-DDX21 or anti-β-actin antibody. Lipo2000, Lipofectamine 2000. (I and J) Representative results, with graphs representing the band intensity ratios of cDDX21/DDX21/β-actin normalized to the control conditions for the RNA (I) and DNA (J) ligand treatment groups. (K) Schematic representation of two transcript isoforms of DDX21. (L to N) HeLa cells were transfected with either an empty vector, Flag–DDX21-X1, or Flag–DDX21-X2. Twenty-four hours after transfection, cells were mock treated or infected with VSV (L), NDV (M), or HSV-1 (N) at an MOI of 1. Cells were harvested at 18 hpi and detected by immunoblot analysis with anti-DDX21, anti-β-actin, or anti-viral-protein (VSV-G, NDV-NP, or HSV-1-gD) antibody.

Interestingly, in both the knockdown and overexpression experiments, we observed the apparent cleavage of DDX21 in the course of VSV infection (Fig. 1A and H; Fig. S1C). To further confirm the cleavage of DDX21 by virus infection, cells were infected by two RNA viruses, VSV and NDV, and one DNA virus, herpes simplex virus 1 (HSV-1), followed by DDX21 detection. As expected, VSV and NDV apparently cleaved DDX21 at 12, 18, and 24 hpi. Full-length DDX21 almost disappeared 18 and 24 h after VSV and NDV infection (Fig. 2A and B). In comparison, HSV-1 only slightly cleaved DDX21, even at the late stage of infection, and the amount of full-length DDX21 was not significantly decreased (Fig. 2C). Statistically, at 18 and 24 hpi, the ratio of DDX21/cleaved DDX21/β-actin was 33 to 564 in VSV- and NDV-infected cells, compared with 2.4 to 2.6 in HSV-1-infected cells (Fig. 2D to F). A549, Huh7, and THP-1 cells were then utilized to test whether virus-triggered DDX21 cleavage was cell type dependent. The results showed that DDX21 was cleaved in A549, Huh7, or THP-1 cells upon VSV or NDV infection (Fig. S2A to D). Additionally, the cleavage of DDX21 was observed in cells infected with Sendai virus (SeV), another RNA virus (Fig. S2E).

10.1128/mBio.01005-21.2 FIG S2 Cleavage of DDX21 by virus infection in various cell types. (A and B) A549 cells were mock treated or infected with VSV (A) or NDV (B) at an MOI of 1. Cells were harvested at 6, 12, 18, and 24 hpi and detected by immunoblot analysis with anti-DDX21, anti-β-actin, or anti-viral-protein (VSV-G or NDV-NP) antibody. (C and D) Huh7 (C) or THP-1 (D) cells were infected with VSV. The virus infection experiments were performed as described above for panel A. (E) HeLa cells were infected with Sendai virus (SeV). The virus infection experiments were performed as described above for panel A. Download FIG S2, TIF file, 0.7 MB. Copyright © 2021 Wu et al. 2021 Wu et al. https://creativecommons.org/licenses/by/4.0/ This content is distributed under the terms of the Creative Commons Attribution 4.0 International license.

Given that DDX21 was cleaved upon RNA and DNA virus infection, and as DDX21 belongs to the RNA helicase family that can bind various RNAs (22), we propose that this cleavage is triggered by virus nucleotides. Two RNA ligands, poly(I·C) and 3p-hpRNA, and two DNA ligands, poly(G·C) and HSV-60, were used to evaluate their role in DDX21 cleavage. As expected, both RNA and DNA ligands cleaved DDX21 (Fig. 2G and H), and a higher cleavage efficiency was observed upon treatment with RNA ligands than upon treatment with DNA ligands (Fig. 2I and J), which is in accordance with the results of virus infection. There are two transcript variants of DDX21, isoform 1 (X1), which encodes full-length DDX21, and isoform 2 (X2), with a shorter N terminus (deletion of residues 1 to 86 [Δ1–86]) than that of X1. Here, we showed that both exogenously expressed DDX21-X1 and -X2 were cleaved upon VSV, NDV, and HSV-1 infection (Fig. 2L to N). Collectively, these results clearly demonstrated that DDX21 was cleaved by virus infection and treatment with RNA/DNA ligands.

### DDX21 was cleaved in a caspase-dependent manner.

**FIG 3:**
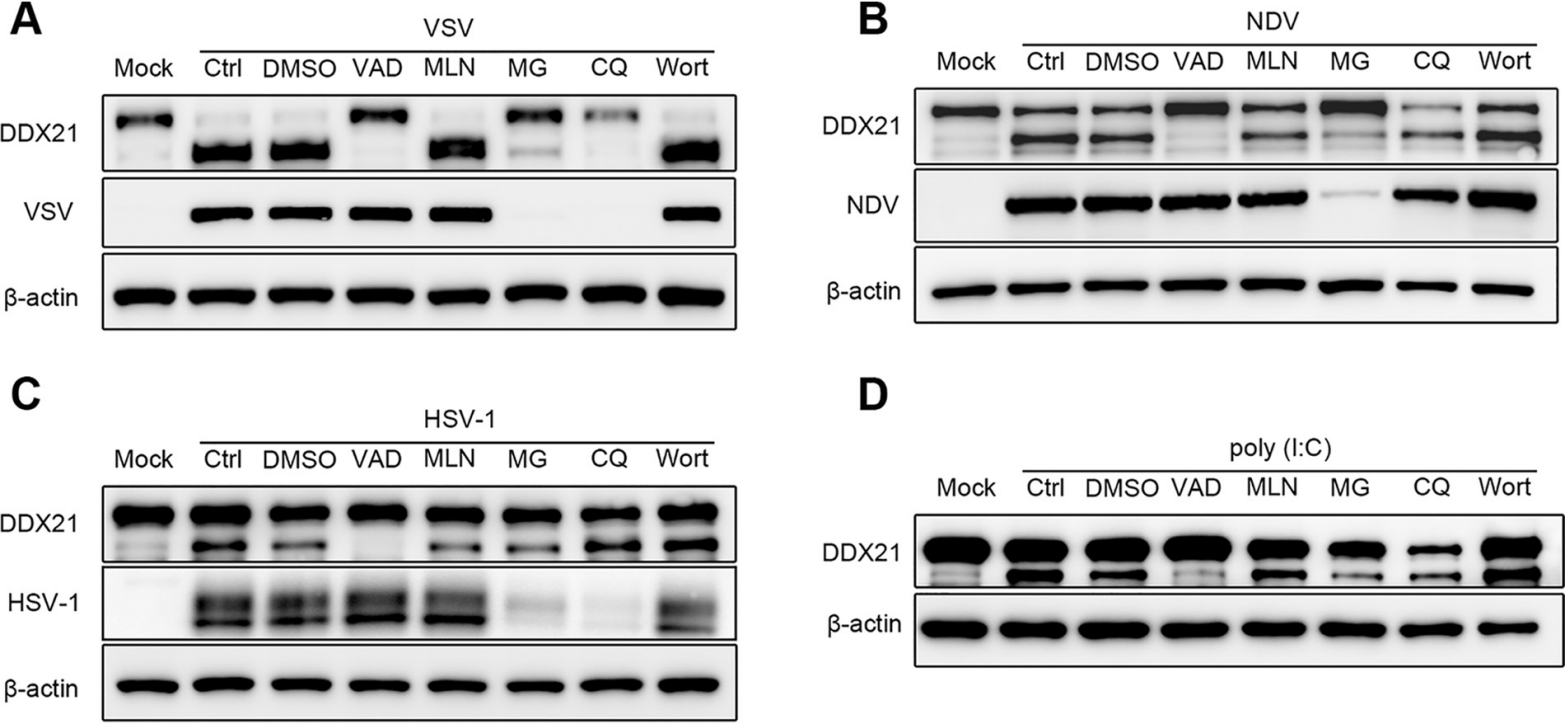
Caspase-dependent cleavage of DDX21 triggered by virus infection or treatment with RNA ligands. (A to C) HeLa cells were mock infected or infected with VSV (A), NDV (B), or HSV-1 (C) at an MOI of 1 and maintained in the presence of the dimethyl sulfoxide (DMSO) control, z-VAD-FMK (VAD), MLN4924 (MLN), MG-132 (MG), CQ, or wortmannin (Wort) for 18 h. Cells were harvested and detected by immunoblot analysis with anti-DDX21, anti-β-actin, or anti-viral-protein (VSV-G, HSV-1-gD, or NDV-NP) antibody. (D) HeLa cells were mock treated or transfected with RNA ligands [poly(I·C)] and maintained in the presence of the DMSO control, z-VAD-FMK, MLN4924, MG-132, CQ, or wortmannin for 18 h. Cells were harvested and detected by immunoblot analysis with anti-DDX21 or anti-β-actin antibody.

To further confirm whether DDX21 was cleaved or degraded upon virus infection and RNA/DNA ligand treatment, cells were treated with the caspase inhibitor carbobenzoxy-valyl-alanyl-aspartyl-[*O*-methyl]-fluoromethylketone (z-VAD-FMK), the neddylation inhibitor MLN4924, the proteasome inhibitor MG-132, and the autophagy inhibitors wortmannin and chloroquine (CQ), followed by virus or treatment with ligand. The results showed that DDX21 cleavage was completely inhibited after treatment with the caspase inhibitor z-VAD-FMK in cells infected with VSV, NDV, and HSV-1 (Fig. 3A to D, lane 4) or treated with the RNA ligand poly(I·C) (Fig. 3D, lane 4). Notably, MG-132 and CQ also seemed to inhibit the cleavage of DDX21, especially in VSV- and NDV-infected cells (Fig. 3A and B, lanes 6 and 7). However, the expression of viral protein was also inhibited after treatment with these two drugs, indicating that the inhibition of DDX21 cleavage may be due to their inhibition of virus replication. In comparison, z-VAD-FMK had no effect on virus replication. These results indicated that DDX21 was cleaved in a caspase-dependent manner.

### Caspase-3/6 cleaved DDX21 at D126 in response to VSV infection.

**FIG 4:**
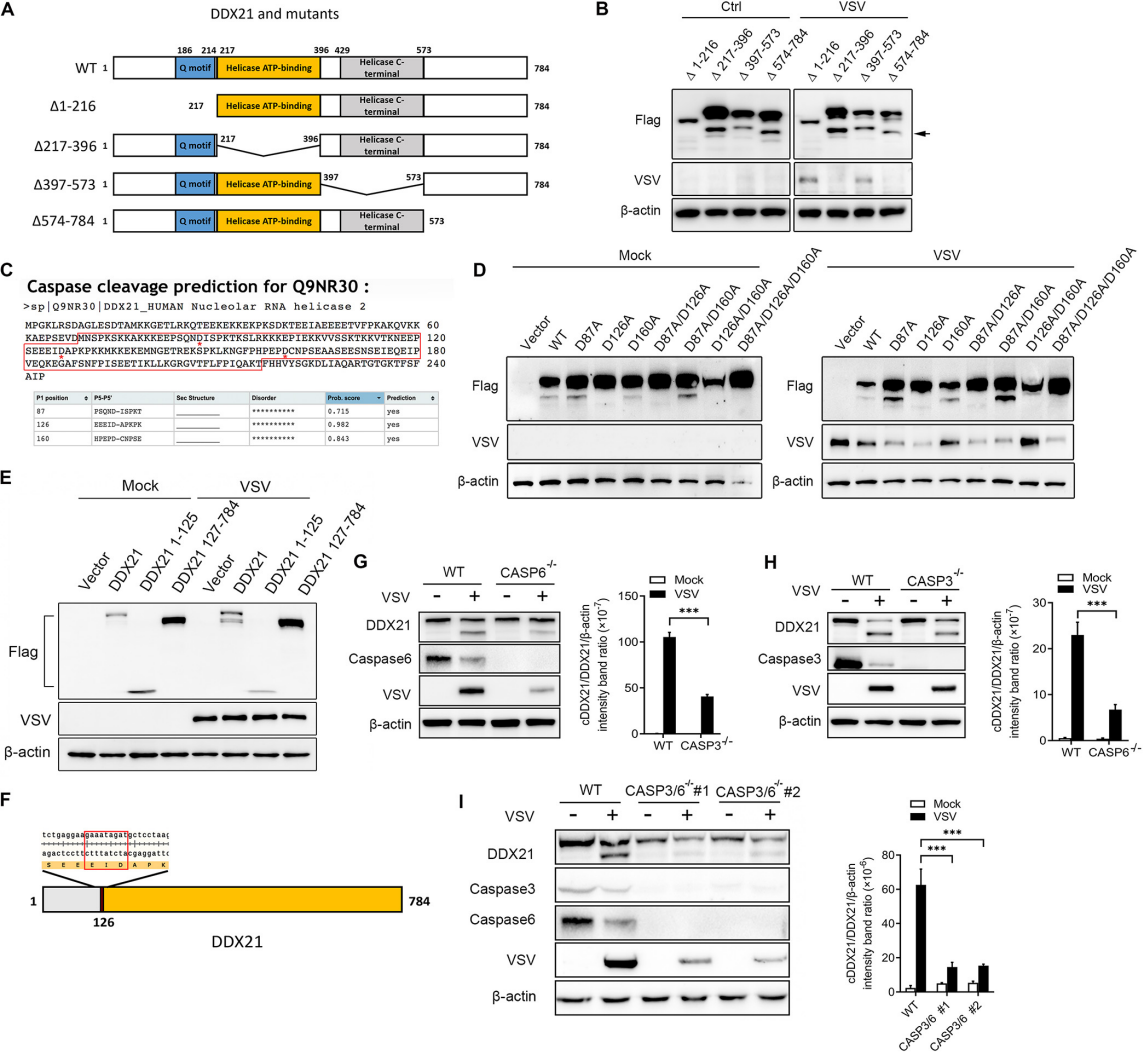
DDX21 was cleaved at D126 by caspase-3/6 in response to VSV infection. (A) Schematic representation of WT DDX21 and its deletion mutants. (B) HeLa cells were transfected with Flag-tagged DDX21 truncates (Δ1–216, Δ217–396, Δ397–573, and Δ574–784). Twenty-four hours after transfection, cells were mock treated or infected with VSV at an MOI of 1. Cells were harvested at 18 hpi and detected by immunoblot analysis with anti-Flag, anti-VSV-G, or anti-β-actin antibody. (C) Prediction results of caspase cleavage sites for DDX21 based on data in CaspDB (http://caspdb.sanfordburnham.org). The red frame indicates aa 87 to 216 of DDX21. The red asterisks indicate the putative caspase cleavage sites. (D) HeLa cells were transfected with either an empty vector or Flag-tagged WT and mutant DDX21 (D87A, D126A, 160A, D87A/D126A, D87A/160A, D126A/D160A, and D87A/D126A/160A). Twenty-four hours after transfection, cells were mock treated or infected with VSV at an MOI of 1. Cells were harvested at 18 hpi and detected by immunoblot analysis with anti-Flag, anti-VSV-G, or anti-β-actin antibody. (E) HeLa cells were transfected with either an empty vector or Flag-tagged WT and truncated DDX21 (1–125 and 127–784). Twenty-four hours after transfection, cells were mock treated or infected with VSV at an MOI of 1. Cells were harvested at 18 hpi and detected by immunoblot analysis with anti-Flag, anti-VSV-G, or anti-β-actin antibody. (F) Schematic representation of amino acids around the cleavage site. The red frame indicates the general motif for caspase cleavage. (G to I) WT and *casp3* (G) and *casp6* (H) knockout and *casp3/6* double-knockout (I) HeLa cells were mock treated or infected with VSV at an MOI of 1. Cells were harvested at 18 hpi and detected by immunoblot analysis with anti-DDX21, anti-caspase-3/6, anti-VSV-G, or anti-β-actin antibody. Representative results are shown, with graphs representing the band intensity ratios of cleaved DDX21 (cDDX21)/DDX21/β-actin normalized to the control conditions.

DDX21 is characterized by several known domains, the Q motif (amino acids [aa] 186 to 214), the helicase ATP binding domain (aa 217 to 396), and the helicase C-terminal domain (aa 429 to 573) (Fig. 4A). To characterize the cleavage sites of DDX21, based on the identified domains, several deletion mutants (Δ1–216, Δ217–396, Δ397–573, and Δ574–784) were generated to test the critical domain involved in DDX21 cleavage. As shown in Fig. 4B, the deletion of residues 1 to 216 abrogated the cleavage of DDX21. Notably, unlike endogenous DDX21 expression, the exogenous expression of DDX21 truncates alone is able to induce cleavage to some extent (Fig. 4B, left). Similar results could be observed with the overexpression of WT DDX21 (Fig. 2H to J). Combined with the results showing that aa 1 to 216 are critical for DDX21 (Fig. 4B) and that aa 1 to 86 are not required for DDX21 cleavage (Fig. 2H to J), the cleavage sites are within aa 87 to 216 of DDX21. The caspase cleavage sites were then predicted by CaspDB (57). The results showed that all three aspartates (Asp [D]), D87, D126, and D160, are the putative caspase cleavage sites (Fig. 4C). Therefore, DDX21 proteins with single, double, and triple mutations of these three Asp sites were generated, followed by transfection and virus infection. The results showed that D126A, but not D87A or D160A, is sufficient and necessary for DDX21 cleavage (Fig. 4D, lanes 4, 6, 8, and 9). The truncates of DDX21 1–125 and 127–784, together with WT DDX21, were transfected into cells to further confirm that cleavage was mediated by D126. As expected, WT, but not 1–125 and 127–784, DDX21 was cleaved upon virus infection (Fig. 4E). It should be noted that based on the molecular weight of DDX21, we proposed that the “cDDX21” that we observed with endogenous cleavage (∼73 kDa) was DDX21 127–784, while the relatively small cleaved DDX21 (∼14 kDa) was degraded upon virus infection (Fig. 4E). Previous reports showed that the cleavage sites specific for caspases had a general motif (31). The motif for DDX21 cleavage is Glu-Ile-Asp (E-I-D), which is the putative substrate for caspase-3 or caspase-6 (Fig. 4F). *casp3^−/−^* and *casp6^−/−^* cells were generated to verify their role in DDX21 cleavage. As expected, the knockout of *casp3* and especially *casp6* significantly inhibited the cleavage of DDX21 (Fig. 4G and H). DDX21 cleavage was almost blocked in *casp3/6* double-knockout cell lines (Fig. 4I). These results indicated that virus infection cleaved DDX21 at D126 via caspase-3/6.

### DDX21 was cleaved and translocated from the nucleus to the cytoplasm in response to virus infection.

**FIG 5:**
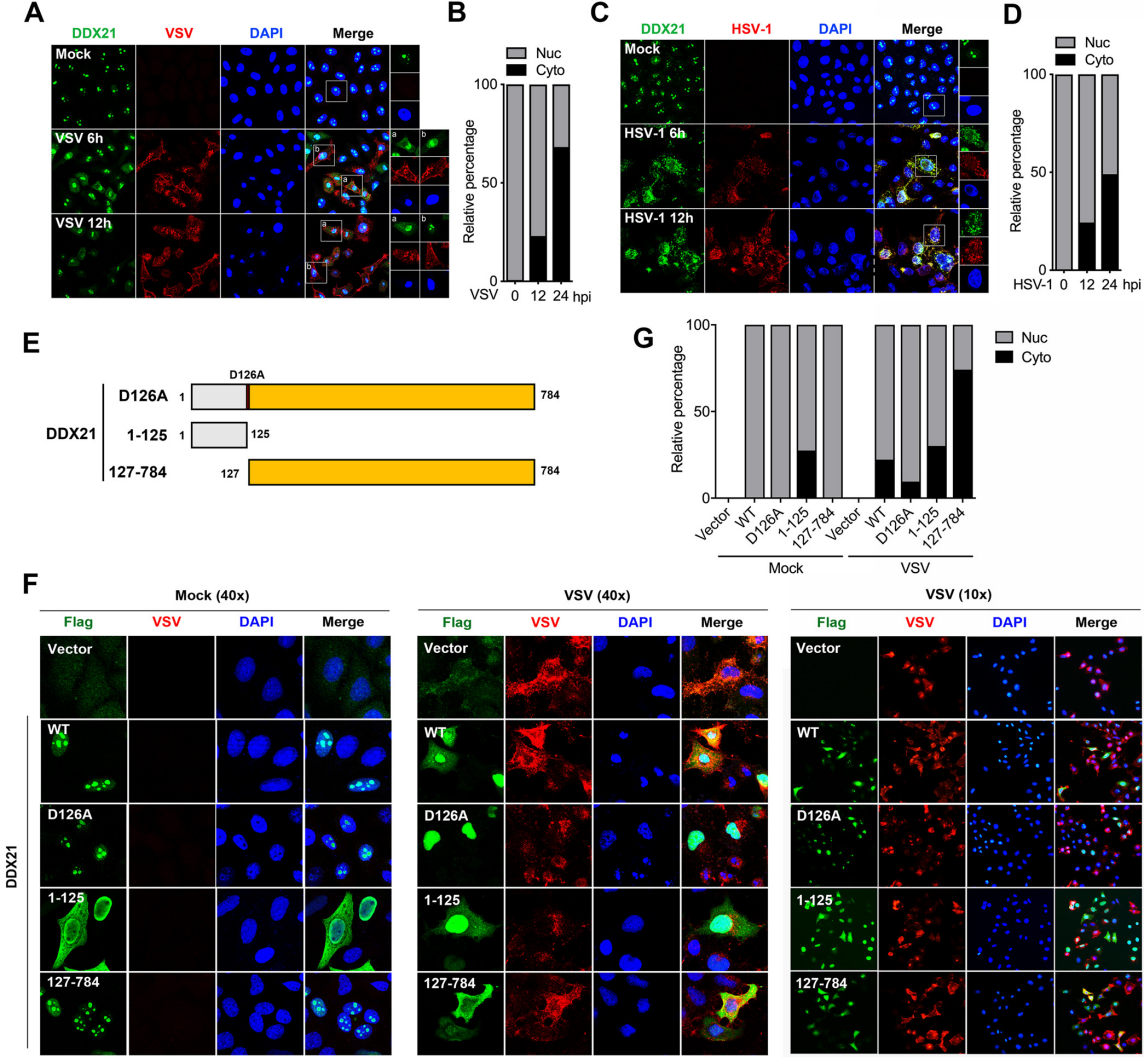
Virus infection triggers the translocation of DDX21 from the nucleus to the cytoplasm, which is dependent on its cleavage. (A and C) HeLa cells were mock treated or infected with VSV (A) or HSV-1 (C) at an MOI of 1. At 6 and 12 hpi, cells were fixed and processed for IF analysis using anti-DDX21 or anti-viral-protein (VSV-G or HSV-1-gD) antibody. Nuclei were stained with 1 μg/ml of DAPI. (B and D) Quantification of the relative percentages of cells with nuclear (Nuc) and cytoplasmic (Cyto) DDX21 staining upon VSV (B) or HSV-1 (D) infection. Ten images in 20 high-power fields (HPFs) were obtained randomly in different fields. (F) HeLa cells were transfected with either an empty vector or Flag-tagged WT, D126A, 1–125, or 127–784 DDX21. Twenty-four hours after transfection, cells were mock treated or infected with VSV at an MOI of 1. At 12 hpi, cells were fixed and processed for IF analysis using anti-Flag or anti-VSV-G antibody. Nuclei were stained with 1 μg/ml of DAPI. (G) Quantification of the relative percentages of cells with nuclear or cytoplasmic DDX21 staining in cells transfected with either an empty vector or Flag-tagged WT, D126A, 1–125, or 127–784 DDX21 upon virus infection. Ten images in 20 HPFs were obtained randomly in different fields.

DDX21 was identified to be a nucleolar helicase that is required for pre-rRNA processing (22, 23, 32). Other reports showed that DDX21 is localized with DDX1, DDX26, and TRIF to sense dsRNA in the cytosol (20). To study whether DDX21 localization was affected by virus infection, cells were infected with VSV and HSV-1, followed by an immunofluorescence (IF) assay (IFA). As expected, endogenous DDX21 was predominantly localized in the nucleolus in mock-infected cells (Fig. 5A and C, top). DDX21 was translocated from the nucleolus to the nucleoplasm and cytoplasm after VSV and HSV-1 infection (Fig. 5A and C, middle and bottom). Statistically, at 12 and 24 hpi, cells with cytoplasmic DDX21 accounted for 23% and 68% of all cells for VSV (Fig. 5B) and 24% and 49% of all cells for HSV-1 (Fig. 5D), respectively, indicating that VSV could induce DDX21 translocation more efficiently than HSV-1. Based on the results above, there are three different forms of DDX21 upon virus infection: full-length DDX21 (D126A), large cleaved DDX21 (127–784), and small cleaved DDX21 (1–125) (Fig. 5E). Next, we evaluated whether the cleavage of DDX21 affected its localization. WT, D126A, 1–125, and 127–784 DDX21, together with the empty vector, were transfected into cells, followed by VSV infection. The results showed that in mock-infected cells, WT, D126A, and 127–784 DDX21 predominantly localized in the nucleolus (Fig. 5F, left). WT and 127–784, but not D126A, DDX21 efficiently translocated from the nucleolus to the nucleoplasm and cytoplasm after VSV infection (Fig. 5F and G), indicating that the blockage of DDX21 cleavage inhibited its translocation. Moreover, the statistical analysis results showed the most efficient translocation of the DDX21 cleaved form (cDDX21) (126–784), further indicating that DDX21 cleavage promotes its translocation from the nucleus to the cytoplasm. Interestingly, 1–125 DDX21 was diffusely localized in the nucleus and cytoplasm in both mock- and VSV-infected cells (Fig. 5F and G). Collectively, these results demonstrated that the virus induced the translocation of DDX21, which is affected by DDX21 cleavage.

### DDX21 cleavage led to the inhibition of the IFN-β signaling pathway.

**FIG 6:**
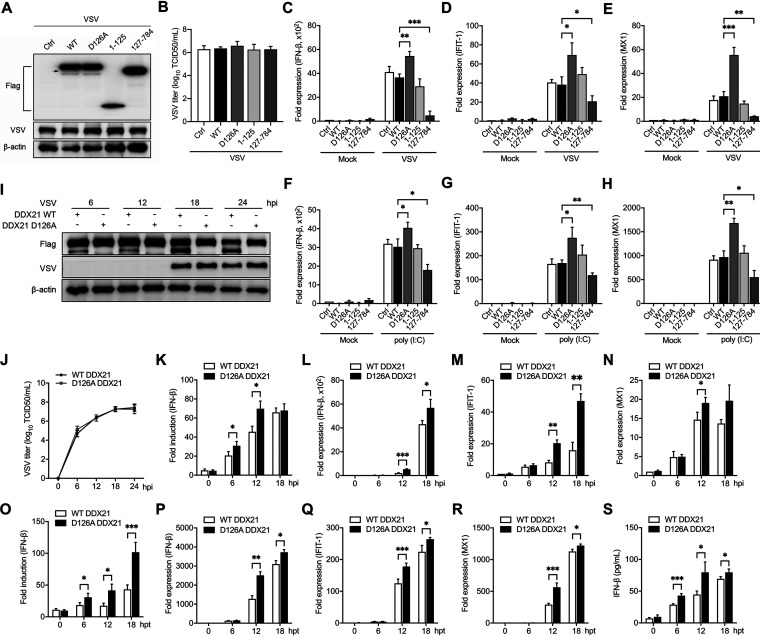
Cleavage of DDX21 impairs IFN-β production. (A) *ddx21^+/−^* HeLa cells were transfected with either an empty vector or Flag-tagged WT, D126A, 1–125, or 127–784 DDX21. Twenty-four hours after transfection, cells were mock treated or infected with VSV at an MOI of 1. At 18 hpi, cells were harvested and detected by immunoblot analysis with anti-Flag, anti-VSV, or anti-β-actin antibody. The arrow indicates cleaved DDX21. (B) Extracellular virus yields in WT and truncated DDX21-transfected *ddx21^+/−^* HeLa cells. (C to E) Virus infection experiments were performed as described above for panel A. Cells were harvested and detected by qRT-PCR with IFN-β (C), IFIT-1 (D), and MX1 (E) primers. (F to H) *ddx21^+/−^* HeLa cells were transfected with either an empty vector or Flag-tagged WT, D126A, 1–125, or 127–784 DDX21. Twenty-four hours after transfection, cells were mock treated or transfected with poly(I·C) (20 μg/ml). Cells were harvested at 12 hpt and detected by qRT-PCR with IFN-β (F), IFIT-1 (G), or MX1 (H) primers. (I) *ddx21^+/−^* cells stably expressing Flag-tagged WT and D126A DDX21 were mock treated or infected with VSV at an MOI of 1. Cells were harvested at 6, 12, 18, and 24 hpi and detected by immunoblot analysis with anti-DDX21, anti-VSV-G, or anti-β-actin antibody. (J) Extracellular virus yields in *ddx21^+/−^* cells stably expressing Flag-tagged WT and D126A DDX21. (K) *ddx21^+/−^* cells stably expressing Flag-tagged WT and D126A DDX21 were cotransfected with IFN-β–Luc and pRL-TK. At 12 hpt, cells were mock treated or infected with VSV at an MOI of 1. Cells were harvested at 6, 12, and 18 hpi and assessed for luciferase activity. The results are presented as relative luciferase activities. (L to N) *ddx21^+/−^* cells stably expressing Flag-tagged WT and D126A DDX21 were mock treated or infected with VSV at an MOI of 1. Cells were harvested at 6, 12, and 18 hpi and detected by qRT-PCR with IFN-β (L), IFIT-1 (M), or MX1 (N) primers. The results are presented as relative luciferase activities. (O) *ddx21^+/−^* cells stably expressing Flag-tagged WT and D126A DDX21 were cotransfected with p-125Luc and pRL-TK. At 12 hpt, cells were mock treated or transfected with poly(I·C) (20 μg/ml). Cells were harvested at 6, 12, and 18 hpt and assessed for luciferase activity. The results are presented as relative luciferase activities. (P to R) *ddx21^+/−^* cells stably expressing Flag-tagged WT and D126A DDX21 were mock treated or transfected with poly(I·C) (20 μg/ml). Cells were harvested at 6, 12, and 18 hpt and detected by qRT-PCR with IFN-β (P), IFIT-1 (Q), or MX1 (R) primers. (S) Poly(I·C) treatments were performed as described above for panel O. Supernatants were collected for quantitation of IFN-β by an ELISA. Data are presented as means from three independent experiments. *, *P* < 0.05; **, *P* < 0.01; ***, *P* < 0.001.

Given that the blockage of DDX21 cleavage inhibits its translocation, we next aimed to explore the effect of DDX21 cleavage on the regulation of innate immunity. *ddx21^+/−^* cells were transfected with WT and truncated DDX21, followed by the detection of virus replication and the IFN-β signaling pathway. Interestingly, transfection of WT and truncated DDX21 did not affect virus replication, evidenced by viral protein expression and titers in the supernatants (Fig. 6A and B). In contrast, although WT DDX21 has no effect, the intact form of DDX21 (D126A) increased the mRNA levels of IFN-β and ISGs (IFIT-1 and MX1) after VSV infection. More importantly, transfection of cDDX21 significantly inhibited the IFN-β signaling pathway (Fig. 6C to E). These results were further confirmed using poly(I·C) as the stimulator (Fig. 6F to H).

To identify whether DDX21 cleavage regulates the IFN-β signaling pathway in a time-dependent manner, *ddx21^+/−^* cells stably expressing Flag-tagged WT and D126A DDX21 were infected by VSV or treated with poly(I·C) and collected at different time points. DDX21 was cleaved in WT but not D126A DDX21-expressing cells. However, no difference was observed in viral protein synthesis between WT and D126A DDX21-expressing cells (Fig. 6I). More importantly, blockage of the cleavage (D126A) significantly increased the IFN-β promoter activity (Fig. 6K and O), mRNA levels of IFN-β and ISGs (IFIT-1 and MX1) (Fig. 6L to N and P to R), and IFN-β production in the supernatants (Fig. 6S), especially at the late stage of virus infection and poly(I·C) treatment. These results clearly indicated that DDX21 cleavage inhibits the IFN-β signaling pathway.

### Cleavage of DDX21 inhibited the formation of the DDX1-DDX21-DHX36 complex.

**FIG 7:**
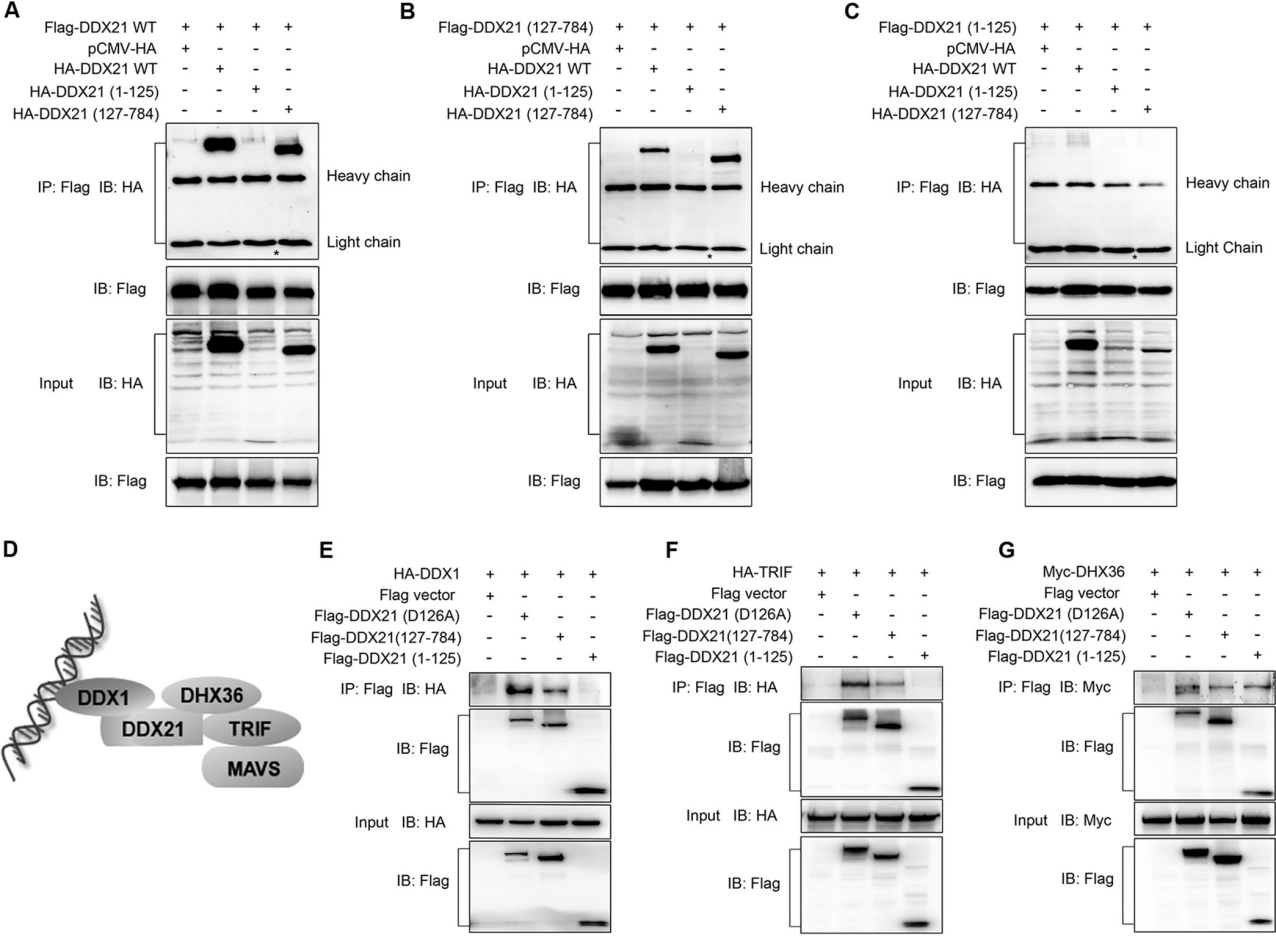
DDX21 cleavage inhibits its interaction with DDX1 and TRIF. (A to C) HeLa cells were transfected with Flag-tagged WT (A), 127–784 (B), and 1–125 (C) DDX21, together with HA-tagged WT, 1–125, or 127–784 DDX21. At 24 hpt, cells were harvested, immunoprecipitated (IP) with anti-Flag antibody, and further detected by immunoblot (IB) analysis with anti-HA or anti-Flag antibody. Expression levels of the proteins were analyzed by immunoblot analysis of the lysates with anti-HA or anti-Flag antibody. The asterisk indicates the predicted size of HA-DDX21 (1–125). (D) The existing model of foreign RNA recognition by the DDX1-DDX21-DDX36 complex. (E to G) HeLa cells were cotransfected with Flag-tagged D126A, 1–125, and 127–784 DDX21 or an empty vector as well as HA-tagged DDX1 (E), HA-tagged TRIF (F), and Myc-tagged DHX36 (G). At 24 hpt, cells were harvested, immunoprecipitated with anti-Flag antibody, and further detected by immunoblot analysis with anti-HA or anti-Flag antibody. Expression levels of the proteins were analyzed by immunoblot analysis of the lysates with anti-HA or anti-Flag antibody.

Given that DDX21 was reported to act as the scaffold protein in the complex of three dsRNA-sensing helicases (DDX1-DDX21-DHX36) (20, 33) (Fig. 7D), we hypothesized that DDX21 cleavage attenuates the inhibition of the IFN-β signaling pathway by the inhibition of the formation of the DDX1-DDX21-DHX36 complex. Since DDX21 was reported to exist as a homodimer (25, 27), the cleavage of DDX21 on its self-interaction was studied first. As shown in Fig. 7A, using full-length DDX21 as the bait, full-length and 127–784, but not 1–125, DDX21 could be immunoprecipitated (Fig. 7A). In accordance, 127–784 DDX21 interacted with 127–784 and full-length, but not 1–125, DDX21 (Fig. 7B). In contrast, 1–125 DDX21 was not immunoprecipitated with WT and truncated DDX21 (Fig. 7C). These results indicated that DDX21 interacted with itself through its C-terminal aa 127 to 784. Next, we evaluated whether DDX21 cleavage inhibits the formation of the DDX1-DDX21-DHX36 complex. The results showed that compared with intact DDX21 (D126A), cDDX21 (127–784) has a low affinity for both DDX1 and TRIF (Fig. 7E and F). In comparison, D126A, 127–784, and 1–125 DDX21 showed binding abilities similar to that of DHX36 (Fig. 7G). Collectively, these results indicated that the cleavage of DDX21 inhibited the formation of the DDX1-DDX21-DHX36 complex and its interaction with the downstream adaptor TRIF.

## DISCUSSION

Intensive functional and structural research over the years has clearly demonstrated that RLRs selectively bind viral RNA ligands and trigger downstream signaling (34). Several other DExD/H box helicases have been implicated in antiviral innate immunity, but fundamental questions such as the precise regulation mechanism of how DExD/H box helicases regulate innate immunity remain to be elucidated. Here, we demonstrated that DDX21 is cleaved at D126 after virus infection and RNA/DNA ligand treatment via the caspase-3/6 pathway, leading to the inhibition of immune responses.

To date, there has been limited research on the cleavage of RNA helicases. Human RNA helicase A, a nuclear helicase that unwinds dsRNA, dsDNA, and RNA-DNA duplexes, was reported to be cleaved by caspase-3 during apoptosis (35). Another study also showed that the CARD-containing helicase (Helicard) is cleaved by caspases upon apoptotic stimuli (36). However, no studies have shown that the cleavage of DNA/RNA helicases is involved in innate immunity and virus infection. Our data showed the obvious cleavage of DDX21 in response to virus infection and treatment with ligands. The fact that three RNA viruses and one DNA virus, together with two RNA and two DNA ligands, trigger various degrees of DDX21 cleavage leads us to speculate that the DDX21 cleavage ability may be shared by most viruses.

The caspase family of cysteine proteases is involved in apoptosis and innate immune signaling (37). In terms of the innate immunity pathway, caspase-1 is one of the most well-studied caspases, which is able to cleave pro-interleukin-1β (pro-IL-1β) and pro-IL-18 and triggers inflammasomes (38). It has also been reported that caspases are involved in the RLR-mediated type I IFN response. For instance, caspase-12 positively modulates the IFN-β signaling pathway by regulating E3 ubiquitin ligase TRIM25-mediated ubiquitination of RIG-I (39). Activated caspase-3/7/9 suppresses mitochondrial DNA-induced stimulator of interferon genes protein (STING)-mediated type I IFN production (40). Here, we showed that the cleavage of DDX21 was completely recovered in the presence of the caspase inhibitor z-VAD-FMK (Fig. 3), and the cleavage was probably mediated by caspase-3/6 (Fig. 4). It should be noted that the knockout of *casp3* and *casp6* did not completely block DDX21 cleavage, indicating that the involvement of other caspases could not be completely excluded. A recent study revealed that caspase-6 cleaves IL-1 receptor (IL-1R)-associated kinase M (IRAK-M) and reduces IκBα degradation, thereby increasing tumor necrosis factor alpha (TNF-α) production (41). The function of caspase-6 in the type I IFN pathway has not been reported. Our study indicated that caspase-3/6 cleaves DDX21 and thus likely regulates the IFN-β signaling pathway.

In a resting state, DDX21, together with its binding partners c-Jun, WDR46, and SIRT7, etc., is localized in the cell nucleolus (23, 32, 42). The nucleolar localization of DDX21 is necessary for its pre-rRNA processing and RNA unwinding (23, 42). Studies have also suggested that DDX21 is involved in innate immunity in the cytoplasm. For example, the DDX1-DDX21-DHX36-TRIF complex may translocate to the mitochondria upon poly(I·C) stimulation (20). Infection of A549 cells with dengue virus causes DDX21 to partially relocate from the nucleus to the cytoplasm (29). Here, we confirmed that DDX21 localized in the cell nucleolus in mock-infected cells. Virus infection effectively triggered DDX21 translocation from the nucleolus to the cytoplasm. In resting cells, WT, D126A, and 127–784 DDX21 showed nucleolar localization. It is interesting that 1–125 DDX21 diffusely localized in the nucleus and the cytoplasm. Previous reports showed that aa 731 to 740 at the C terminus of DDX21 interacted with c-Jun, and the depletion of c-Jun promotes DDX21 translocation from the nucleolus to the nucleoplasm (23). Therefore, it is possible that the deletion of the C terminus of DDX21 abolishes the interaction with its binding partner and thus alters its nucleolar localization. Our results showed that (i) the blockage of DDX21 cleavage inhibits DDX21 translocation and (ii) the most efficient translocation of cDDX21 occurs upon virus infection. These results collectively indicated that cleavage of DDX21 promotes its translocation from the nucleus to the cytoplasm.

Various DDX and DHX helicases play important roles in maintaining the stability of the cell genome (22, 42, 43). To date, the knockout of *ddx21* has never been reported in cells or mice. Studies on other DDXs have also shown that certain DDXs are critical for mouse growth, and knockout of the *ddx* gene results in early embryonic lethality (44, 45). Therefore, DDX21 may also be critical for cell and mouse survival. Nevertheless, DDX21 expression was significantly inhibited in *ddx21^+/−^* cells. Previous studies and our results demonstrated that the depletion of DDX21 significantly inhibited type I IFN production (20), suggesting that DDX21 positively regulates innate immunity. Interestingly, the depletion of DDX21 also impaired virus replication. Considering that DDX21 is a multifunctional protein that also plays an important role in maintaining the stability of the cell genome (27, 42, 46), it may also influence virus replication in ways other than innate immunity. Nevertheless, it is undoubtful that DDX21 *per se* plays a positive role in innate immunity. Most importantly, the cleavage of DDX21 inhibits innate immunity but does not affect virus infection, which leads us to speculate that the cleavage of DDX21 was driven by the host for a late counterregulatory effect to temper immune responses.

**FIG 8:**
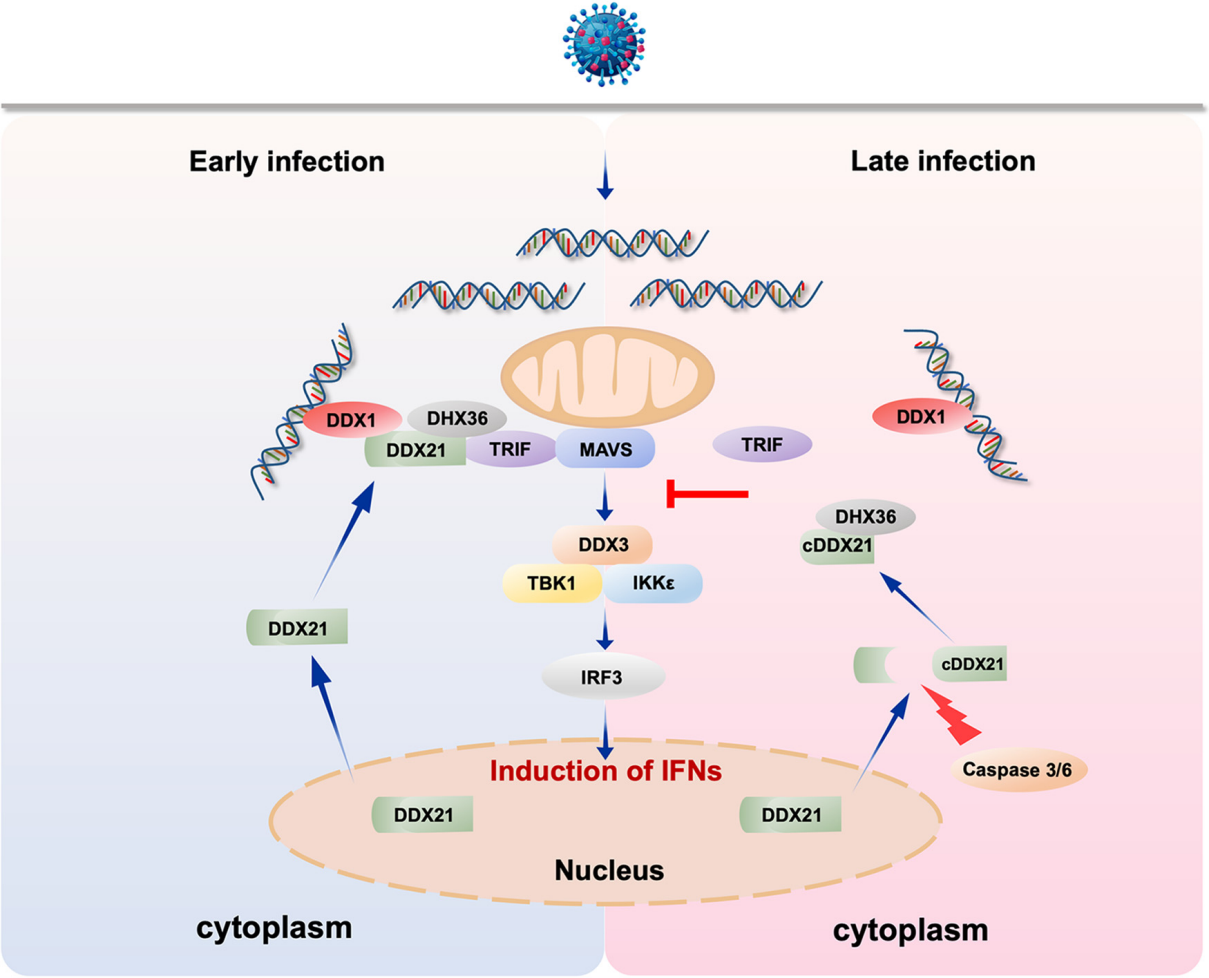
Proposed model for the regulation of innate immunity by DDX21 cleavage during virus infection. The double-edged-sword role of DDX21 in the regulation of innate immunity upon virus infection is depicted. At the early stage of infection, foreign RNA is recognized by DDX1 and recruits DDX21 and DHX36 to form a complex to mediate downstream antiviral innate immunity. At the late stage of infection, to avoid an excessive immune response, the host promotes DDX21 cleavage via caspase-3/6. Cleaved DDX21 tends to translocate from the nucleus to the cytoplasm. DDX21 cleavage reduces the interaction between upstream DDX1 and downstream TRIF and therefore suppresses signal transduction for a late counterregulatory effect to temper immune responses.

Although type I IFN is widely reported to play an essential role against viral infection, the aberrant production of cytokines leads to unexpected pathological consequences in a variety of autoimmune diseases (47, 48). Therefore, the balance between these key pathways is essential for immune homeostasis. Indeed, the DNA sensor cGAS has been reported as a key driver of lethal autoimmune disease in the Trex1-deficient mouse model of Aicardi-Goutieres syndrome (AGS) (49). The importance of excess RLR-dependent signaling, which leads to an IFN signature in the pathogenesis of many autoimmune diseases such as AGS and systemic lupus erythematosus, has also been clarified (50). These reports highlight the importance of fine-tuning the regulation of the type I IFN signaling pathway. Here, we provide several lines of evidence to demonstrate that the host promotes DDX21 cleavage to temper immune responses: (i) cleavage of DDX21 was observed at the late stage of infection; (ii) cleavage was a universal phenomenon not only for virus but also for RNA and DNA mimics; (iii) the blockage of DDX21 (D126A) cleavage increases IFN production, and cleaved DDX21 (aa 127 to 784) inhibits IFN production; (iv) the cleavage of DDX21 did not affect virus replication at the late stage of infection; and (v) the cleavage of DDX21 inhibited the formation of the DDX1-DDX21-DHX36 complex. Collectively, from our original and additional data, we inferred that the host promotes DDX21 cleavage via caspase-3/6 to suppress DDX1-DDX21-DHX36 complex formation for a late counterregulatory effect to temper immune responses (Fig. 8). An improved understanding of these processes could shed light on the causes of infectious disease and, plausibly, immune disorders involving excessive inflammatory immune activities.

## MATERIALS AND METHODS

### Reagents and antibodies.

The caspase inhibitor z-VAD-FMK (catalog number C1202; Beyotime, Nantong, China) was used at a concentration of 50 μM. The neddylation inhibitor MLN4924 (catalog number S7109; Selleckchem, Houston, TX, USA) was used at a concentration of 1 μM. The proteasome inhibitor MG-132 (catalog number S1748; Beyotime) was used at a concentration of 20 μM. The autophagy inhibitors wortmannin (catalog number W1628; Sigma-Aldrich, St. Louis, MO, USA) and chloroquine (CQ) (catalog number C6628; Sigma-Aldrich) were used at 300 nM and 25 μM concentrations, respectively. Poly(I·C) (catalog number tlrl-pic), 3p-hpRNA (catalog number tlrl-hprna), HSV-60 (catalog number tlrl-hsv60n) and poly(dG·dC) (catalog number tlrl-pgcn) were purchased from InvivoGen (San Diego, CA, USA). Rabbit monoclonal anti-DDX21 (catalog number ab182156), anti-caspase-6 (catalog number ab185645), mouse monoclonal anti-vesicular stomatitis virus glycoprotein (VSV-G) (catalog number ab50549), and rabbit polyclonal anti-lamin B1 (catalog number ab16048) antibodies were purchased from Abcam (Cambridge, MA, USA). Rabbit polyclonal anti-herpes simplex virus 1 glycoprotein D (HSV-1-gD) (catalog number NB600-516) was purchased from Novus Biologicals (Littleton, CO). Rabbit polyclonal anti-Sendai virus (catalog number PD029C1) was purchased from MBL (Nagoya, Japan). Rabbit polyclonal anti-caspase-3 (catalog number GTX110543) was purchased from GeneTex (Irvine, CA). Mouse monoclonal anti-Flag (catalog number F1804), antihemagglutinin (anti-HA) (catalog number H9658), and anti-β-actin (catalog number A1978) antibodies were purchased from Sigma-Aldrich. Rabbit monoclonal anti-phospho-TANK binding kinase 1 (p-TBK1) (catalog number 5483) and anti-TBK1 (catalog number 3013) were purchased from Cell Signaling Technology (Beverly, MA, USA). Monoclonal antibody against NDV nucleoprotein (NDV-NP) was prepared in our laboratory (51). For the immunofluorescence assays, mouse monoclonal anti-HSV-1/2 gE was purchased from Santa Cruz Biotechnology (Dallas, TX, USA), and mouse polyclonal anti-VSV-G antibodies (catalog number ab1874) were purchased from Abcam. IFN-β was measured with an enzyme-linked immunosorbent assay (ELISA) kit (catalog number 41410; PBL Assay Science, Piscataway, NJ).

### Cell cultures and virus.

HeLa, A549, HEK-293T, Vero, Huh7, and THP-1 cells were purchased from the American Type Culture Collection (ATCC). These cells were maintained in Dulbecco’s modified Eagle’s medium (DMEM) supplemented with 10% fetal bovine serum (FBS) (Thermo Fisher Scientific, Waltham, MA, USA). The NDV Herts/33 strain was obtained from the China Institute of Veterinary Drug Control (Beijing, China). HSV-1 was kindly provided by Yasushi Kawaguchi (University of Tokyo, Japan), and VSV was provided by Jianchao Wei (Shanghai Veterinary Research Institute, China). Sendai virus (SeV) was provided by Quan Zhang (Yangzhou University, China). HSV-1 and VSV titers were determined as the median tissue culture infective doses (TCID_50_s) on Vero cells.

### Plasmids.

Flag-tagged DDX21-X1 (Flag-DDX21) and Flag-DDX21-X2 were constructed by inserting the open reading frame (ORF) of human DDX21 isoform 1 (GenBank accession number NM_004728.4) and isoform 2 (accession number NM_001256910.2) into plasmid p3XFLAG-CMV-14 (Sigma-Aldrich), respectively. Flag-tagged deletion constructs (Δ217–396 and Δ397–573) and point mutants of DDX21 (D87A, D126A, 160A, D87A/D126A, D87A/160A, D126A/D160A, and D87A/D126A/160A) were generated by site-directed mutagenesis, as described previously (52, 53). Flag- and HA-tagged wild-type and truncates of DDX21 (1–125, 127–784, Δ1–216, and Δ574–784) were constructed by inserting the indicated sequences into p3XFLAG-CMV-14 (Sigma-Aldrich) and pCMV-HA (Promega), respectively. HA-tagged DDX1 and HA-tagged TRIF were constructed by inserting the indicated sequences into pCMV-HA (Promega). Myc-tagged DHX36 was constructed by inserting the ORF of DHX36 into pCMV-Myc (Promega). pHAGE-WT and -D126A DDX21 were constructed by inserting Flag-tagged WT and D126A DDX21 into pHAGE-bsd, which was constructed based on pHAGE-puro (Addgene plasmid 118692). The primer sequences for plasmid construction are listed in Table S1 in the supplemental material. The IFN-β promoter luciferase reporter was kindly provided by Takeshi Fujita (Kyoto University, Japan).

10.1128/mBio.01005-21.4 TABLE S1 Primers and small interfering RNAs (siRNAs) used in this study. Download Table S1, DOCX file, 0.5 MB. Copyright © 2021 Wu et al. 2021 Wu et al. https://creativecommons.org/licenses/by/4.0/ This content is distributed under the terms of the Creative Commons Attribution 4.0 International license.

### Cell transfection, luciferase assay, and gene knockdown.

Cells were transfected using FuGENE HD (Promega, Madison, WI, USA) or Lipofectamine 2000 (Thermo Fisher Scientific) according to the manufacturer’s instructions. For the luciferase assay, cells were cultured in 24-well plates and cotransfected with 100 ng of a firefly luciferase reporter (IFN-β–Luc) and 10 ng of the constitutive *Renilla* luciferase reporter pRL-TK. Luciferase activity was measured at 24 h posttransfection (hpt). Lentiviral short hairpin RNAs (shRNAs) for targeting endogenous DDX21 (5′-CCCATATCTGAAGAAACTATT-3′) were purchased from Gene Pharma (Shanghai, China). To generate a DDX21 stable knockdown cell line, HeLa cells were infected with lentiviral shRNA for DDX21 (shDDX21) and selected by puromycin as described previously (54).

### Immunofluorescence assay.

HeLa cells were washed in phosphate-buffered saline (PBS), fixed in 4% neutral formaldehyde, and then permeabilized with 0.5% Triton X-100 in Tris-buffered saline with Tween 20 (TBST) for 10 min. After blocking in TBST with 3% bovine serum albumin, the cells were incubated with primary antibody for 1 h at 37°C. The cells were washed three times with TBST and incubated with secondary antibody. Subsequently, the cells were washed and incubated with another primary antibody, followed by incubation with secondary antibody. Next, the cells were washed again and incubated with 0.5 μg/ml 4′,6-diamidino-2-phenylindole (DAPI). The coverslips were washed and visualized using a Zeiss LSM 880 confocal microscope (Carl Zeiss, Jena, Germany).

### Nucleocytoplasmic separation assay.

Nuclear extracts were prepared using NE-PER nuclear and cytoplasmic extraction reagents (catalog number 78833; Thermo Fisher Scientific) according to the manufacturer’s instructions.

### Immunoblotting and coimmunoprecipitation.

Immunoblotting was performed as described previously (51). Briefly, cells were lysed in cell lysis buffer containing a protease inhibitor cocktail (Merck Millipore, Darmstadt, Germany). The lysates were denatured and then subjected to sodium dodecyl sulfate-polyacrylamide gel electrophoresis (SDS-PAGE) and immunoblotting and quantified using ImageJ software. For coimmunoprecipitation, HeLa cells were transfected with expression vectors for 24 or 36 h and lysed with cell lysis buffer (150 mM NaCl, 50 mM Tris-HCl [pH 8.0], 5 mM EDTA, 0.5% NP-40) containing 1 mM phenylmethylsulfonyl fluoride (PMSF) and protease inhibitors (Merck Millipore). The lysates were centrifuged at 12,000 × *g* for 10 min and precipitated with anti-Flag antibody, in conjunction with protein G agarose beads (Thermo Fisher Scientific), overnight at 4°C. The beads were washed with lysis buffer four times, eluted with SDS loading buffer by boiling for 10 min, and then subjected to immunoblotting.

### Quantitative real-time PCR.

Quantitative real-time PCR (qRT-PCR) was performed as described previously (55). Briefly, total RNA was extracted, reverse transcribed to cDNA, and subjected to qRT-PCR analysis using Premix Ex *Taq* reagents (TaKaRa, Dalian, China). Primers were referenced from previous reports (55) (Table S1). The relative abundance of mRNAs was calculated using the comparative threshold cycle (ΔΔ*C_T_*) method (56). All experiments were carried out in triplicate.

### Generation of knockout cells.

The CRISPR/Cas9 system was used to generate *ddx21* and *casp6* knockout cells. The guide RNA (gRNA) specific for the *ddx21* and *casp6* genes was designed using the online CRISPR design tool (http://crispr.mit.edu/). The gRNA oligonucleotides were annealed and cloned into the pGK1.1 vector (Geneloci, Nanjing, China). HeLa cells were electrotransfected with the plasmid at 550 V, with 1 pulse. After 24 h of electrotransfection, the supernatants were replaced with DMEM plus 10% FBS supplemented with 1 μg/ml puromycin (Merck Millipore) for 24 h. The pooled (mixed clones) cells were preliminarily sequenced and validated. The candidate positive clones were then subcloned onto 96-well plates using the limiting-dilution method. The genomic region surrounding the CRISPR target site was amplified by PCR using the check primers. The primer sequences for single guide RNAs (sgRNAs) and check primers are listed in Table S1. The clones were sequenced to ensure the frameshifting mutation of both alleles of the established cell line. Except for PCR verification, the cells were rechecked by immunoblotting using DDX21 antibody. *casp3^−/−^* HeLa cells were purchased from EdiGene Inc. (Beijing, China) and verified by immunoblotting. Caspase-3/6 double-knockout cells were constructed by transfecting *casp6* sgRNA into *casp3^−/−^* HeLa cells as described above.

### Generation of cells stably expressing WT and mutant DDX21.

HEK-293T cells were transfected with pHAGE-WT and -D126A DDX21, together with two packaging plasmids, pMD2.G (Addgene plasmid 12259) and psPAX2 (Addgene plasmid 12260). The supernatants were collected at 60 hpt, centrifuged at 5,000 rpm for 10 min, and filtered. The lentivirus supernatants supplemented with 5 μg/ml Polybrene (Sigma-Aldrich) were added to the *ddx21^+/−^* HeLa cells. The cells were horizontally centrifuged at 1,000 rpm for 90 min and incubated at 37°C for 48 h. The supernatants were then replaced with 10% FBS supplemented with blasticidin (Merck Millipore) for 72 h. The cells were subcloned by limiting dilution and confirmed by immunoblotting. The primer sequences for stable expression are listed in Table S1.

### Statistical analysis.

Data are expressed as means ± standard deviations. Significance was determined with two-tailed independent Student’s *t* test (*P < *0.05) between two groups. One-way analysis of variance (ANOVA) was followed by Tukey’s test to compare multiple groups (>2).

10.1128/mBio.01005-21.3 FIG S3 Subcellular distribution of WT and truncated DDX21 by a nucleocytoplasmic separation assay. *ddx21^+/−^* HeLa cells were transfected with either an empty vector or Flag-tagged WT, D126A, 1–125, or 127–784 DDX21. Twenty-four hours after transfection, cells were mock treated or infected with VSV at an MOI of 1. At 18 hpi, cells were harvested for a nucleocytoplasmic separation assay and detected by immunoblot analysis with anti-Flag, anti-β-actin, or anti-lamin B1 antibody. Download FIG S3, TIF file, 0.5 MB. Copyright © 2021 Wu et al. 2021 Wu et al. https://creativecommons.org/licenses/by/4.0/ This content is distributed under the terms of the Creative Commons Attribution 4.0 International license.
