# Supplementary figures and images for: Caspase-Dependent Cleavage of DDX21 Suppresses Host Innate Immunity
Source: mBio. 2021 Jun 14;12(3):e01005-21. doi: 10.1128/mBio.01005-21 (PMC8262918; doi:10.1128/mBio.01005-21)

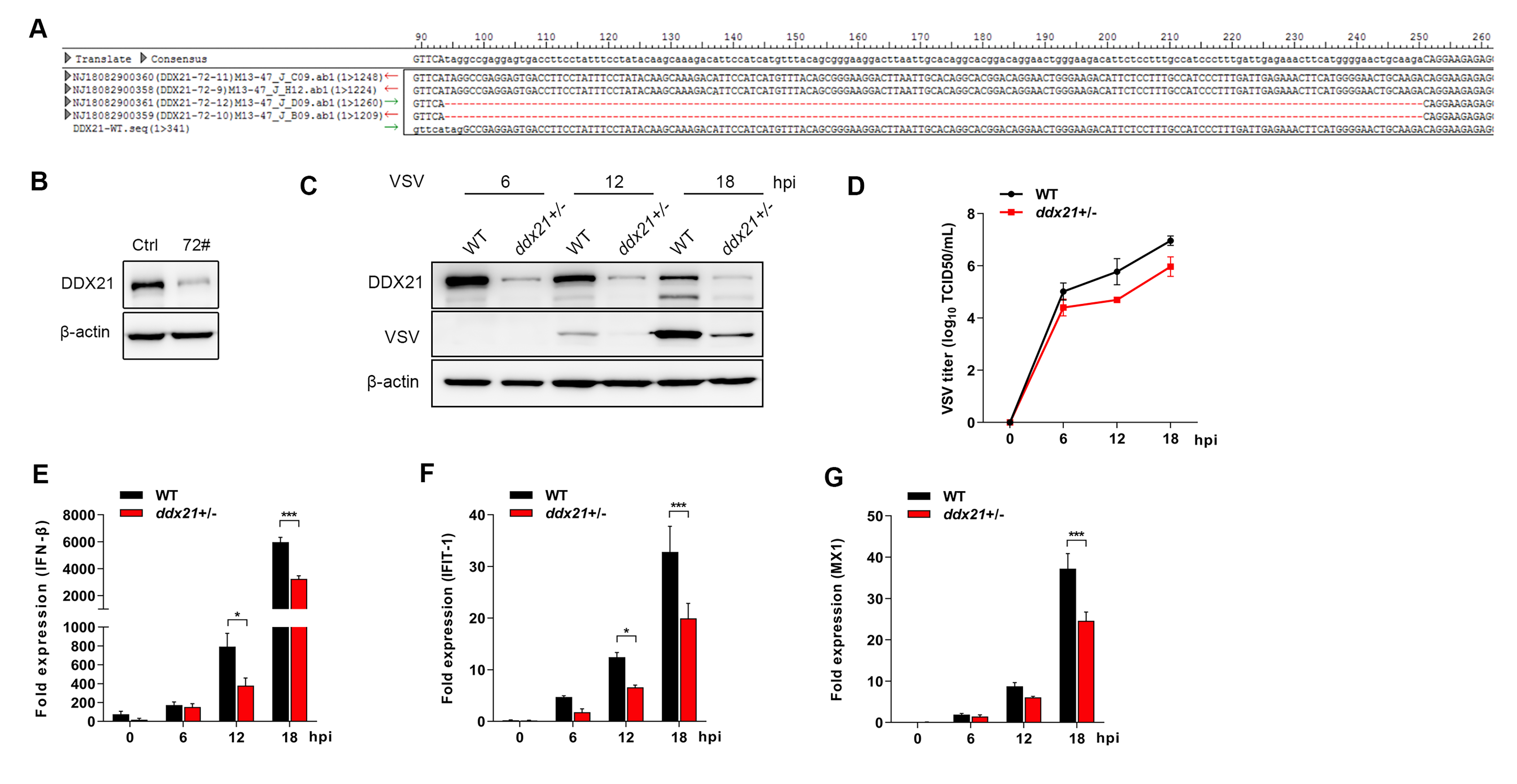

Supplement: FIG S1 [file mbio.01005-21-sf001.tif]

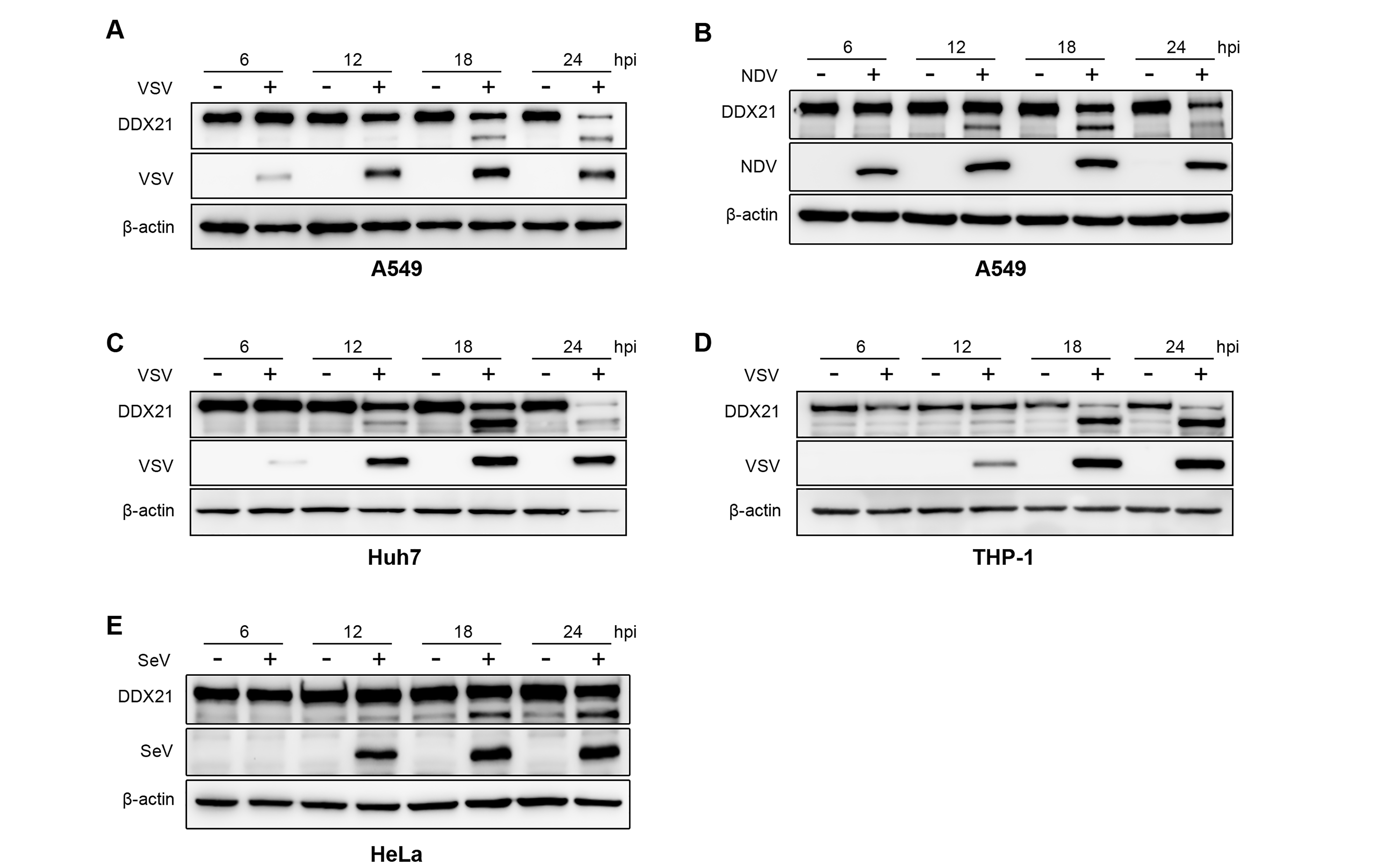

Supplement: FIG S2 [file mbio.01005-21-sf002.tif]

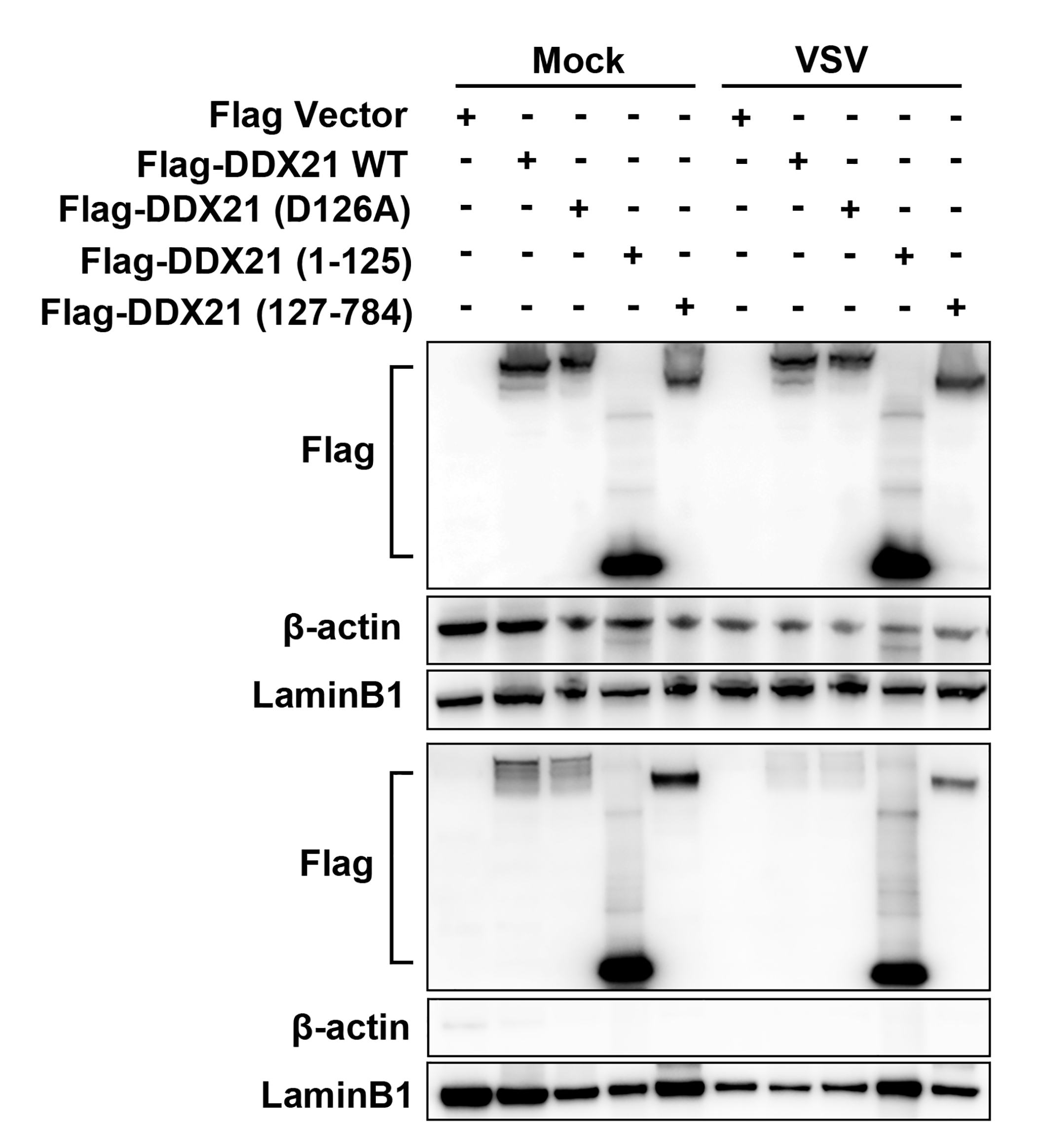

Supplement: FIG S3 [file mbio.01005-21-sf003.tif]
