# Supplementary material for: Caspase-Dependent Cleavage of DDX21 Suppresses Host Innate Immunity
Source: mBio. 2021 Jun 14;12(3):e01005-21. doi: 10.1128/mBio.01005-21 (PMC8262918; doi:10.1128/mBio.01005-21)
Supplement: TABLE S1 [file mbio.01005-21-st001.docx]

Table S1 Primers used in this study

| Primers | Application | Sequence (5’-3’) |
| --- | --- | --- |
| Flag-DDX21 X1-F | Cloning | CGGGGTACCAATGCCGGGAAAACTCCG |
| Flag-DDX21 X1-R |  | CGCGGATCCTTGACCAAATGCTTTACTGAAACTC |
| Flag-DDX21 X2-F |  | CGGGGTACCAATGAATTCTCCTAAATCCAAAAAGG |
| Flag-DDX21 X2-R |  | CGCGGATCCTTGACCAAATGCTTTACTGAAACTC |
| HA-DDX21-F |  | TGGCCATGGAGGCCCGAATTCGGATGCCGGGAAAACTCCGTAGTGAC |
| HA-DDX21-R |  | CTTATCATGTCTGGATCCCCGCGGCCGCTTATTGACCAAATGCTTTACTGAAACTCCGC |
| Flag-DDX21 ∆1-216-L | Truncates and deletion mutants | CGGGGTACCATTCCATCATGTTTACAGCGGG |
| Flag-DDX21 ∆1-216-R |  | CGCGGATCCTTGACCAAATGCTTTACTGAAACTC |
| Flag-DDX21 ∆217-396-L |  | agcaaagacagtggacctga |
| Flag-DDX21 ∆217-396-R |  | tcaggtccactgtctttgct |
| Flag-DDX21 ∆397-573-L |  | atatgaacaggcttccagca |
| Flag-DDX21 ∆397-573-R |  | tgctggaagcctgttcatat |
| Flag-DDX21 ∆574-784L |  | CGGGGTACCAATGCCGGGAAAACTCCG |
| Flag-DDX21 ∆574-784-R |  | CGCGGATCCTTTTATTATTTCTGTTGCAGAAGGAAC |
| Flag-DDX21 1-126-L |  | CGGGGTACCCCGATGCCGGGAAAACTCCGT |
| Flag-DDX21 1-126-R |  | CGCGGATCCGCGTATTTCTTCCTCAGAAGG |
| Flag-DDX21 127-784-L |  | CGGGGTACCATGGCTCCTAAGCCCAAGAAG |
| Flag-DDX21 127-784-R |  | CGCGGATCCGCGTTGACCAAATGCTTTACT |
| HA-DDX21 1-126-L |  | TGGCCATGGAGGCCCGAATTCGGATGCCGGGAAAACTCCGTAGTGAC |
| HA-DDX21 1-126-R |  | CTTATCATGTCTGGATCCCCGCGGCCGCTTATATTTCTTCCTCAGAAGGCTCCTCATTTTTT |
| HA-DDX21 127-784-L |  | TCTTATGGCCATGGAGGCCCGAATTCGGatgGCTCCTAAGCCCAAGAAGATGAAGAAAGA |
| HA-DDX21 127-784-R |  | CTTATCATGTCTGGATCCCCGCGGCCGCTTATTGACCAAATGCTTTACTGAAACTCCGC |
| Flag-DDX21 D87A-L | Site mutations | TCTCAAAATGCCATTTCTCCTAAAACCAAAAGTTTGAGAAAGAA |
| Flag-DDX21 D87A-R |  | AGGAGAAATGGCATTTTGAGATGGCTCCTCTTTCTTTTTTG |
| Flag-DDX21 D126A-L |  | GAAGAAATAGCTGCTCCTAAGCCCAAGAAGATGAAGAAAGA |
| Flag-DDX21 D126A-R |  | CTTAGGAGCAGCTATTTCTTCCTCAGAAGGCTCCTCATTTTTT |
| Flag-DDX21 D160A-L |  | CCTGAACCGGCCTGTAACCCCAGTGAAGCTGCCAGTG |
| Flag-DDX21 D160A-R |  | GGGGTTACAGGCCGGTTCAGGATGAGGAAATCCATTCT |
| pHAGE-DDX21  (WT/D126A)-L | Stable expressing | ttcaggtgtcgtgaagcggccgcATGCCGGGAAAACTCCGTAGTGAC |
| pHAGE-DDX21  (WT/D126A)-R |  | cgttaggggggggggtctagaCTACTTGTCATCGTCATCCTTGTA |
| *ddx21*-sgRNA | Knockout | TTCATGGGGAACTGCAAGAC |
| *ddx21*-check F |  | GGACCAGAAGCATGAGTAGT |
| *ddx21*-check R |  | TATGTACACAATGCCCAAGT |
| *casp6*-sgRNA |  | GCTAACAGTTGACACTATAAAGG |
| *casp6*--check F |  | CCACAAATTTTCCTCCTCCC |
| *casp6*--check R |  | AACGGCCTGTCCAATTACTG |
| qIFIT-1 F | qRT-PCR | GCCATTTTCTTTGCTTCCCCT |
| qIFIT-1 R |  | TGCCCTTTTGTAGCCTCCTTG |
| qβ-actin F |  | GATCTGGCACCACACCTTCT |
| qβ-actin R |  | GGGGTGTTGAAGGTCTCAAA |
| qIFN-β L |  | ACGACAGCTCTTTCCATGA |
| qIFN-β R |  | AGCCAGTGCTCGATGAATCT |
| qMX1 F |  | tgcgcccctgcatcgacct |
| qMX1 R |  | gtttcttcagtttcagcacca |
